# A 3-year prospective study of the incidence of gastric ulcers in pigs slaughtered at Base Abattoir in Rwanda

**DOI:** 10.14202/vetworld.2017.873-879

**Published:** 2017-08-07

**Authors:** Borden Mushonga, Bernard Yabaragiye, Erick Kandiwa, Gervais Habarugira, Alaster Samkange

**Affiliations:** 1 Department of Biomedical Sciences, School of Veterinary Medicine, Faculty of Agriculture and Natural Resources, University of Namibia, P. Bag 13301, Pioneerspark, Windhoek, Namibia; 2 School of Animal Sciences and Veterinary Medicine, College of Agriculture, Animal Sciences and Veterinary Medicine, University of Rwanda, P. O. Box 57 Nyagatare, Rwanda; 3 Department of Veterinary Clinical Studies, School of Veterinary Medicine, Faculty of Agriculture and Natural Resources, University of Namibia, P. Bag 13301, Pioneerspark, Windhoek, Namibia

**Keywords:** Base Abattoir, gastric ulcers, pigs, Rwanda

## Abstract

**Aim:**

Determination of the incidence of gastric ulcers in pigs slaughtered at Base Abattoir in Rwanda.

**Materials and Methods:**

Stomachs from all 5040 pigs that were slaughtered at Base Abattoir in Rwanda from August 2012 to August 2015 were examined for the presence and location of gastric ulcers. The results of the inspections were recorded and analyzed. Statistical analysis for Chi-square values was performed using the Software Package for Social Sciences version 16.0. The Z test for comparison of proportions was used and p<0.05 was considered significant.

**Results:**

Overall as well as per district, significantly more male pigs than female pigs were slaughtered (p<0.05). The incidence of gastric ulcers in slaughter pigs was 12.86%. The incidence of gastric ulcers in males (13.36%) was not significantly different (p>0.05) from that in female pigs (12.84%) in all three districts. A significantly greater proportion of these ulcers (69.03%) was located in the esophageal region than in the glandular region of the stomach (30.97%) in slaughtered males (p<0.05). A significantly greater proportion of these ulcers (79.59%) was located in the esophageal region than in the glandular region of the stomach (20.41%) in slaughtered females (p<0.05). The overall incidence of esophageal ulcers (9.44%) in the slaughtered pigs was significantly (p<0.05) higher than that of glandular region ulcers (3.41%). Pigs with carcass weight over 60 kg showed a significantly (p<0.05) higher (44.44%) incidence of ulcers than those between 40 and 60 kg (33.33%) and those below 40 kg (22.22%).

**Conclusions:**

It was concluded that the incidence of gastric ulceration in slaughter pigs at Base Abattoir was not associated with source (district) or sex of pigs but was associated with the carcass weight.

## Introduction

A gastric ulcer is a mucosal defect in which the entire epithelial thickness, down to or through the basement membrane has been lost [1]. The condition is quite common in man, dogs, cats’ foals, cattle and is commonplace in pigs [1-3]. The problem is responsible for significant revenue losses in modern pig production enterprises [4-6]. Understanding the factors that influence the development of ulcers is therefore critical in designing management practices that minimize the development of ulcers in pigs [7].

Gastric ulceration in pigs has been observed and recorded as early as the mid 1940’s [8]. Most research regards gastric ulcers as the most common pathological finding in the stomach of pigs at slaughter and thus an important cause of sudden death from gastrointestinal bleeding in live pigs from some herds [7,9].

In pigs, ulceration can occur in the glandular and nonglandular (esophageal) parts of the stomach [7,10] although it occurs more commonly in the later (nonglandular or esophageal) region of the stomach [7,11]. On the other hand, ulceration of the glandular region, that is, the cardiac, fundic and pyloric regions [12] is regarded as less common and has normally been associated with systemic infections [13].

Several studies have evaluated the association between age, sex, and breed with the occurrence of gastric ulcers in pigs and no statistically significant findings were reported for age and sex although the problem was reported to be higher in sows than in finishing pigs [7] and higher in barrows than in gilts [10]. However, conflicting results have been reported for breed [7,14-17].

The prevalence of esophageal ulcers in finishing pigs varies widely across different studies, ranging from 2.3% observed in Norway [18] to 5.1% in South of Africa [19], and 6.4% reported by other workers [20]. The prevalence figures as high as 15% have been reported in the last 20-25 years [9], and there is a general feeling that the prevalence of ulcers has been increasing over the same period [20]. More recently, the ulcer prevalence figures have been adopted as a measure of animal welfare standards [20,21]. The prevalence and economic impact of gastric ulceration in pigs of Rwanda are unknown.

Nutritional (dietary form), management, and infectious risk factors have been identified and studied extensively in relation to gastric ulcers [22,23]. The chief culprits among dietary forms that had a tendency to increase gastric ulceration were fine grinding [24], expansion, hydrothermal treatment and pelleting of feed [7,25]. According to Nguyen *et al*. [26], feeding crumbled feed may have an effect in reducing ulceration. Restricted feeding, large farm size, water quality, slatted floor type have featured prominently in literature as causes of gastric ulceration [24]. Holding pigs overnight before slaughter can also cause preslaughter stress which can also lead to the development of gastric ulcers [20]. However, reports of ambient temperatures have produced conflicting results. Supplying straw to pigs has been found to reduce the incidence of ulcers in pigs [21,27-29]. There are also conflicting reports on the association between growth performance and occurrence of gastric ulcers [30,31]. Many studies have reported a causative association between *Helicobacter* infections and gastric ulceration [24,32]. It has also been suggested that quality of water plays an important role in the causation of gastric ulcers and can act as a source of *Helicobacter* species or gastrospirillum [7,33]. Other factors that have also been mentioned include housing without litter and the use of nonsteroidal anti-inflammatory drugs (NSAIDs) [11].

Although the pathogenesis of gastric ulceration is known to be multifactorial and complex, it essentially involves a tilt in the balance between defensive factors and the buffering system resulting in prolonged activation of pepsinogens and changes in mucus composition [10,24]. Gastric acid production, pepsin, ethanol, bile salts, and drugs such as NSAIDs have all been identified as aggravating factors while the mucus-bicarbonate layer, prostaglandins, cellular degeneration and mucosal blood flow were identified as defensive factors [15]. It would appear that the production of urease by *Helicobacter* species results in the hydrolysis of urea to carbon dioxide and ammonia. Ammonia then neutralizes the acidity in the stomach and allows colonization of gastric mucosa by the bacteria [23]. Urease also abates the inflammatory processes that result in the development of gastric ulcers [31]. Ulcer complications can include perforations leading to severe peritonitis, blood loss, and even death [34].

Deaths occur mostly at the grower-finisher stage and have always had a significant economic impact on operations [4,9]. Different researchers have documented contradictory evidence on the impact of gastric ulceration on live weight gain of pigs [35-37]. However, the cross-sectional nature of these studies precludes a proper interpretation of the temporal relationships between these phenomena. Economics and welfare concerns justify the monitoring of pig populations to determine the prevalence and severity of these stomach lesions [4].

A number of techniques including postmortem examination of abattoir specimens by gross inspection [38] as well as by histology [31], endoscopy [30], gastrocamera photography [39], and clinical biochemistry have been used in the diagnosis of gastric ulcers in pigs.

S-methylmethionine sulfonium chloride has been used at 200 mg/kg for supplementation and as prevention or therapy for esophagogastric ulcers in pigs with no significant benefits [39]. The efficacy of melatonin in the treatment of gastric ulcers in pigs has also been demonstrated [40]. There are no commercially viable treatments for commercial pigs although proton pump inhibitors can be used in pet pigs and very valuable breeding animals [41].

According to Monteiro [7], management strategies to prevent ulceration in pigs require an in-depth understanding of the nutritional and management factors involved in the etiopathogenesis of gastric ulceration in pigs.

The aim of this study was to explore gastric ulceration and associated factors in slaughter pigs from three districts of Northern Rwanda.

## Materials and Methods

### Ethical approval

Since only abattoir samples were used for the study, ethical approval was therefore not necessary for this kind of the study.

### Study area

The study was conducted at Base Abattoir located in Rulindo district, in the Northern Province of Rwanda. The owners of the abattoir were notified of the aims, scope, duration, and procedures of this study and provided the researchers with written consent before its commencement. The abattoir’s catchment area exclusively includes Rulindo, Gicumbi, and Kicukiro districts. During the duration of the study, the abattoir slaughtered 612, 1404 and 3024 pigs from Gicumbi, Kicukiro and Rulindo districts, respectively. In the course of the study, the researchers visited randomly selected pig farms in the three districts in order to familiarize themselves with the scale of operations and the husbandry practices of the farmers. Pig farming in these districts was primarily practiced by smallholder farmers.

### Inspection at abattoir

A 3-year prospective study was done on all 5040 pigs slaughtered at Base Abattoir from August 2012 to August 2015. The breeds of pigs slaughtered were of the Large White, Landrace and Landrace-Large white cross varieties. Ethical slaughter process was carried out in the usual format of evisceration, splitting, washing followed by postmortem inspection. The stomachs of all animals were opened along the greater curvature using a pair of scissors, emptied, washed with running tap water and then inverted to allow visual inspection of the gastric mucosa by veterinary pathologists in the research team. The presence and location (esophageal or glandular) of gastric ulcers were noted and recorded.

### Statistical analysis

Statistical analysis for Chi-square values was performed using the Software Package for Social Sciences version 16.0. The Z test for comparison of proportions was used and p<0.05 was considered significant.

## Results

There was a significant difference (p<0.05) between the total numbers of female and male pigs which were slaughtered at Rulindo abattoir (Table 1), with more males slaughtered than females.

**Table-1:** Total number of pigs that were slaughtered at Base Abattoir categorized according to district of origin and sex.

District	Male pigs slaughtered	Female pigs slaughtered	Total	Proportion (%)	p value
Gicumbi	396	216	612	12.14	0.00*
Kicukiro	1080	324	1404	27.86	0.00*
Rulindo	1908	1116	3024	60.00	0.00*
Total	3384	1656	5040	100.00	-

* Significant difference between number of males and females slaughtered (p<0.05)

There was no significant difference (p>0.05) in the incidence of gastric ulcers between male and female pigs that were slaughtered (Tables-2 and 3).

**Table-2:** Occurrence of gastric ulcers in slaughtered pigs according to district and sex.

District	Male pigs with gastric ulcers	Female pigs with gastric ulcers	Total number of pigs with ulcers	p value
Gicumbi	76	32	108	0.50^#^
Kicukiro	124	56	180	0.15^#^
Rulindo	252	108	360	0.15^#^
Total	452	196	648	-

# No significant difference in the occurrence of gastric ulcers between male and female pigs (p>0.05)

**Table-3:** Overall occurrence of gastric ulcers in slaughtered pigs according to sex.

District	Slaughtered pigs with gastric ulcers	Slaughtered pigs without gastric ulcers	Proportion of pigs with gastric ulcers (%)	Total
Male pigs	452	2932	13.36	3384
Female pigs	196	1460	11.84	1656
Total	648	4392	12.86	5040

In male pigs, there was a significant difference in the location of the gastric ulcers (p<0.05); there were more ulcers in the esophageal region compared to the glandular region (Table 4). The picture was the same for females pigs (Table 5).

**Table-4:** Occurrence of esophageal and glandular gastric ulcers in slaughtered male pigs according to district.

District	Ulcers in esophageal region	Ulcers in glandular region	Total	p value
Gicumbi	60	16	76	0.01*
Kicukiro	84	40	124	0.05*
Rulindo	176	76	252	0.00*
Total	312	140	452	0.00*

* Significant difference in the location of gastric ulcers (p<0.05)

**Table-5:** Occurrence of esophageal and glandular gastric ulcers in slaughtered female pigs according to district.

District	Ulcers in esophageal region	Ulcers in glandular region	Total number of female pigs with ulcers	p value
Gicumbi	28	4	32	0.03*
Kicukiro	44	12	56	0.03*
Rulindo	84	24	108	0.00*
Total	156	40	196	0.00*

* Significant difference in the location of gastric ulcers (p<0.05)

In total, there were significantly more (p<0.05) esophageal ulcers than glandular ulcers (Table 6).

**Table-6:** Overall occurrence of esophageal and glandular gastric ulcers according to district.

District	Ulcers in esophageal region	Proportion of ulcers in slaughtered population (%)	Ulcers in glandular region	Proportion of ulcers in slaughtered population (%)	p value
Gicumbi	88	1.74	20	0.40	0.03*
Kicukiro	128	2.54	52	1.03	0.03*
Rulindo	260	5.16	100	1.98	0.00*
Total	476	9.44	172	3.41	0.00*

* Significant difference in occurrence of esophageal and glandular region gastric ulcers (p<0.05)

The incidence of esophageal region ulcers in the >60 kg category was significantly higher than that in the 40-60 kg category (p<0.05), which in turn was significantly higher than that in <40 kg category (p<0.05) (Table 7).

**Table-7:** Location of pig gastric ulcers according to carcass weight category.

Carcass weight category	Pigs with esophageal region ulcers	Pigs with glandular region ulcers	Total number of male pigs with ulcers	Proportion of pigs with ulcers (%)
<40 kg	112	32	144	22.22
40-60 kg	160	56	216	33.33
>60 kg	196	92	288	44.44
Total	468	180	648	100.00

The results in Table 8 illustrate the distribution of pig gastric and esophageal ulcers across weight categories at slaughter. Statistical analysis of the results in Table 8 using cross-tabulated Z scores and p values showed that the incidence of glandular region ulcers in the >60 kg category was significantly higher than that in the 40-60 kg category (p<0.05) which in turn was significantly higher than that in <40 kg category (p<0.05). Overall, the incidence of gastric ulcers in the >60 kg category was significantly higher than that in the 40-60 kg category (p<0.05) which in turn was significantly higher than that in <40 kg category (p<0.05).

**Table-8:** Distribution of pig ulcers according to weight categories.

Categories of lesions	<40 kg category	40-60 kg category	>60 kg category	Grand total
Pigs with esophageal ulcers	112	160	196	468
Pigs without esophageal ulcers	2021	1440	1111	4572
Category total	2133	1600	1307	5040
Pigs with glandular region ulcers	32	56	92	180
Pigs without glandular region ulcers	2101	1544	1215	4860
Category total	2133	1600	1307	5040

## Discussion

An overall incidence of porcine gastric esophageal ulcers of 9.44% in this study was higher than the incidence recorded in other African studies [19,42]. However, a much higher percentage was reported in Canada (15.5%), USA (16.4%), Portugal (18.7%), United Kingdom (19.6%), and Australia (31%) [7,9,36,37,39,43]. The overall occurrence of porcine gastric glandular ulcers of 3.41% in this study appears to show an increase from the 0.19% observed in the 1970s [44], the 1.2% recorded in the early 60’s [45], and the 2.1% recorded in the 70’s [45]. However, there was also another study in which incidences ranging from 12% to 29% were also reported [46]. It is tempting to conclude that the 3.41% occurrence figure, shown by our study, is a reflection of an upward trend reported by previous workers [44,45]. The fact that pig feed processing techniques have achieved progressively smaller particle sizes over the past four decades suggests that pigs have been at progressively higher risk for ulceration over the same period. There have been suggestions that technology and industrialization which have seen an increase from the 60’s to the 80’s may be responsible for the concomitant rise in ulceration in pigs [7].

The ulcers in glandular region have been associated with systemic diseases such as salmonellosis, erysipelas or hog cholera infections [45,47]. The first possible explanation of higher occurrence of glandular ulcers in this study was the relatively low availability (and even total lack) of veterinary services and disease control in the three districts that provided animals slaughtered at Base Abattoir. The second possible explanation was the poor level of hygiene resulting from lack of clean water sources and inadequate waste disposal facilities observed in most farms visited in the course of the study. In an earlier study in Australia, animals receiving dam water had a higher prevalence of esophageal gastric ulcers than those from farms using water from a borehole [37]. These Australian researchers blamed high levels of bacteria for reduced water quality resulting in bacterial infections predisposing animals to esophageal gastric ulcers [37]. As in humans, the colonization of the stomach by organisms of the *Helicobacter*-type was suggestively connected with the development of gastric ulcers in pigs [14,33,48-50]. The third observation made by the authors on visiting sample pig farms in the three districts was a realization that piggeries in Kicukiro and Rulindo were much larger operations but without corresponding improvements in clean water supply, husbandry techniques and facilities.

The significant variation in the occurrence of esophageal gastric ulcers between districts was indicative of the predisposing factors of the district of origin. A similar pattern was reported in Portugal showing significant differences in the occurrence of esophageal gastric ulcers between nine different farms under the study [7]. In one study, significant differences in the percentage of gastric ulcers between two farms were demonstrated [45]. Differences in the prevalence of esophageal gastric ulcers between herds and in distinct Australian states were also observed [37]. The prevalence of esophageal gastric ulcers in Victoria (53%) was significantly higher than in Western Australia (30%) or Queensland (7%). Large herd sizes and their negative impact on management and nutritional practices were cited as possible causative factors for such a pattern. This study noticed the same trend whereby farmers increased herd sizes so as to increase incomes without necessarily improving husbandry facilities and practices.

Findings of no significant difference in the frequency of esophageal gastric ulcers between males and females in this study were supported by findings from other studies [35,37,51]. The risk factors associated with the occurrence of gastric ulcers have been largely documented and evaluated [4,37]. This study noted that the main risk factors associated with the occurrence of gastric ulcers in pigs were water, diet (main and supplement food), and hygiene (waste management).

Feeding pigs with finely ground feeds was associated with a higher incidence of ulcers in such herds [52-54]. Other studies showed feeding pigs with pelleted feeds was also associated with a higher incidence of ulcers [10,18,24,43,54,55,56]. Feed restriction for economic reasons or as a feeding practice was suggested as a possible stress factor resulting from lower dietary fiber and resulting in gastric ulceration in some studies [37]. Breed was also shown as a predisposing factor in the incidence of porcine gastric ulcers when a higher incidence of ulcers (29%) in the Duroc breed was compared to the lower incidence (12%) in the Yorkshire breed [46]. Other studies did not yield consistent results to prove such an association between genetic background and gastric ulcers [35,47,54].

## Authors’ Contributions

BM contributed in designing of the study, supervision of the project, and writing of the final version of the manuscript. BY did the administration of the survey, collection and examination of abattoir samples and writing of the draft manuscript. EK did the designing of the experiment, the statistical analysis and the final manuscript write up and editing. GH did the data analysis, the write up of the draft and editing of the final version of the manuscript. AS did the designing of the studies, write-up of the draft, editing the final version and coordinated the publication of this manuscript. All authors read and approved the final manuscript.
